# Developmental Drift and the Role of Wnt Signaling in Aging

**DOI:** 10.3390/cancers8080073

**Published:** 2016-08-02

**Authors:** Jan Gruber, Zhuangli Yee, Nicholas S. Tolwinski

**Affiliations:** 1 Yale-NUS College, Singapore 138527, Singapore; Gruber@yale-nus.edu.sg; 2 Department of Biochemistry, Yong Loo Lin School of Medicine, National University of Singapore, Singapore 117597, Singapore; yee.zhuangli@u.nus.edu; 3 Department of Biological Sciences, National University of Singapore, Singapore 138615, Singapore

**Keywords:** wnt, aging, developmental drift

## Abstract

Population aging is a public health problem affecting the majority of the developed world. As populations age, the incidence of degenerative diseases increases exponentially, leading to large increases in public spending on healthcare. Here we summarize recent findings on the developmental drift theory of aging, and the links that have been established between aging and the Wnt signaling pathways. We focus on insights derived from model organisms connecting the evolutionary basis of aging and the link to developmental programming.

## 1. Introduction: Wnt and Aging

Wnt signals regulate a wide range of cellular functions throughout development and adulthood. To date, most studies have concentrated on the developmental functions of the various Wnt signaling pathways, stem cells, and crucially, on cancer causing mutations in adults. The Wnt genes encode an evolutionarily conserved group of ligands that activate a network of signaling pathways. Wnts have been linked to cell proliferation, cell fate choice, apoptosis and cell polarity during embryonic development, and stem cell maintenance in adults [1,2,3]. The main Wnt pathway is the “canonical” pathway, which relies on stabilization of β-catenin leading to transcriptional activation. Wnt binding to its receptors inactivates the destruction complex. In the absence of Wnt, this destruction complex phosphorylates β-catenin marking it for proteasome-mediated degradation preventing β-catenin from entering the nucleus and affecting transcription [4,5]. Mutations in pathway components lead to major developmental defects in a variety of model systems, and are causal in human cancers [6]. The specifics of Wnt signaling mechanisms are reviewed in detail elsewhere in this issue, instead, this review will focus on an emerging view of the interplay between aging and development and especially the functions of developmental signaling pathways and how they relate to aging in adult animals [7].

The focus on aging is motivated by the fact that populations in many developed nations today are aging at an unprecedented rate. These changes are due to the combined effect of a positive increase in life expectancy and falling fertility rates. For example, in Singapore average life expectancy in 1970 was under 73, increasing to 85 in 2010. Fertility rates over the same period have dropped from 3.1 to 1.15 a level significantly below the replacement rate of ~2.1 [8,9]. In his seminal 1952 lecture on aging, entitled “An Unsolved Problem of Biology”, Sir Peter Medawar pointed out that an exponential increase in morbidity and mortality with age is a defining property of the aging process [10]. Specifically, within an aging population, morbidity and mortality increase exponentially with the percentage of aged people in the population. Cancer, cardiovascular disease, Alzheimer disease and other neurodegenerative diseases as well as general frailty are all strongly age-dependent, where age is one of the main, if not the single most important risk factor for these conditions [11,12,13]. Another defining feature of the aging process (even of so called “healthy aging”) is a decline in homeostatic capacity, that is, a pronounced reduction in the ability to mount robust stress responses needed to recover fully from physiological perturbations [14]. Population aging is thus one of the major social, scientific and economic challenges of our time. Aging research, therefore, ought to develop preventative interventions leading to an increased health span reducing age-related morbidity [12,15,16,17,18].

Wnt’s roles in specific diseases are described elsewhere in this issue, and here we focus on the fact that most age-related diseases depend on biological age as their principal risk factor suggesting that their etiology is intricately linked to the aging process itself [12,18,19,20]. This view implies that a better understanding of the basic mechanisms of aging may be required to delay or prevent age-dependent diseases [12,19]. An important first step is to identify basic mechanisms and regulators of aging and lifespan determination. These mechanisms must be “public”, or conserved across large evolutionary distances from worms to mammals, and not “private” mechanisms that are specific to individual species likely not applying to human aging [21,22]. The question regarding the degree of conservation of basic aging mechanisms has direct implications to human health, not least because most mechanistic aging research is carried out in model organisms. Identifying evolutionarily conserved genes, pathways and mechanisms involved in regulating lifespan and life history is therefore a central goal of aging research.

## 2. Wnt, a Developmental Signaling Pathway

Developmental signaling pathways are key factors directing embryonic development and are conserved amongst many species. Mutations in these signaling pathways, such as TGFβ, Hedgehog, Wnt and others lead to a variety of developmental defects in animals [6]. The fact that these pathways are highly conserved across species has greatly facilitated their study as model organisms have been used with great success to identify components and organize them into pathways. The Wnt pathway is one such highly conserved module with myriad organisms from Drosophila to humans encoding a number of Wnt genes, and the mechanism of signal transduction especially in the canonical pathway being conserved.

Relatively few studies have looked at the homeostatic functions of Wnt pathways, except as they relate to stem cell maintenance [2]. A number of studies have suggested that Wnt signaling has positive effects on health during aging. As organisms age, proliferating cells stop dividing and stem cells are lost. Wnt signaling can counteract this as it maintains stem cells in their niches with positive effects on neurogenesis and bone regeneration [23,24,25]. Wnt’s positive effects can be countered by inhibition of the pathway leading to detrimental effects typical of aging related diseases like osteoporosis and Alzheimer’s disease [26]. More recent findings, however, also point to detrimental effects of Wnt signaling as organisms age. For example, one study found that muscle degeneration in older mice could be traced to an increase of circulating Wnt ligand in the bloodstream of older individuals, where this signal prompted cells that would otherwise become new muscle cells to differentiate into fibroblasts instead [27]. Another study, looked at mice that age prematurely due to a mutation in the *klotho* gene finding that *klotho* appears to be a secreted Wnt antagonist whose absence leads to ectopic Wnt signaling and a premature aging phenotype [28]. These contradictory results show Wnt has both positive and negative consequences on aging making Wnt’s role in aging both complex and controversial [26].

To resolve these contrasting findings, the most promising recent approach has combined a powerful aging and developmental model organism by studying Wnts in *C. elegans*. Budovskaya and Kim have published a series of studies linking the developmental drift theory (see below) of aging with late Wnt expression. Their findings are of particular interest, as they show how Wnts can be both detrimental and helpful for the aging process in worms depending on the specific Wnt ligand, timing and tissue involved. For instance, suppression of *Mom-2/Wnt* shortens lifespan significantly, while suppression of *lin-44/Wnt* can be beneficial [7]. As there are only five Wnt genes in *C. elegans*, and worms only live for a short time, these experiments were done relatively easily, whereas such work would have been difficult to perform in mice where there are 19 Wnt genes and lifespans last years. The finding that different Wnts can have such disparate effects on aging should explain why studies in vertebrates have found such complex and contrasting effects.

## 3. Developmental Drift Theory of Aging

So how can these complex and apparently contradictory roles of Wnt signaling be understood? One of the maxims of modern biology states: "nothing in biology makes sense except in the light of evolution [29].” Modern evolutionary theories of aging are based on the key insight, attributed to Haldane and Medawar, that because in the wild most individuals die of extrinsic causes (e.g., predation, infection or accident) before late-acting mutations or genes can take effect, such late acting mutations or genes are under far weaker selection than those that take effect early in life. Because extrinsic mortality is generally high in nature, the force of natural selection declines rapidly with age [30]. Genotypes that, though detrimental late in life, actually have early beneficial functions and confer early benefits [30,31]. Such trade-offs in terms of late costs for early benefits are known as antagonistic pleiotropy [30,31]. Genes showing antagonistic pleiotropy are actively selected for, because the beneficial phenotype promotes survival during a time when the force of selection is high, while the detrimental effects become apparent only at an age when the force of selection is low [10]. As a consequence, genes or gene mutations that have late acting, detrimental phenotypes cannot be efficiently eliminated by natural selection and may therefore accumulate or be actively selected for in natural populations making aging a maladaptive trait that serves no purpose in itself, rather it emerges only as a side effect of antagonistic pleiotropy. Evolution selects for early fitness, and the selection force declines rapidly as organisms age [22]. It is likely, therefore, that there are no “gerontogens”, or genes that have evolved specifically to cause aging or promote age-dependent decline in organismal function [14]. Instead, genes affecting aging rate, longevity assurance and health span are selected based on their early, advantageous effects on evolutionary fitness, even when incurring late costs in terms of detrimental aging phenotypes [22,30,32]. This idea explains why many “aging” pathways, or pathways that when mutated extend lifespan, are negative regulators of homeostasis and stress response mechanism. These genes were not originally selected because of their (detrimental) role on aging and longevity but as part of pleiotropic tradeoff between resource investment into early maintenance and stress response and other, more immediately beneficial processes, such as fecundity and factors governing speed of development [33]. This means that lifespan-extending mutations should typically be expected to be associated with some evolutionary cost or tradeoff. Long-lived mutants are thus expected to be rapidly outcompeted by short-lived wild type animals, at least in the wild [31].

This expectation is borne out by the genes and pathways that have been identified as major, evolutionarily conserved determinants of aging and longevity. The first-ever single gene mutation shown to dramatically extend lifespan (in any organism) was the *age-1* gene, identified in the 1980s in *C. elegans* [34,35,36]. The *age-1* gene is part of the insulin-like growth factor (IGF) axis, and mutations in this gene cause lifespan extensions of up to 10 fold in *C. elegans* [37]. The realization that modulation of a single pathway could dramatically extend lifespan was surprising, but even more so was the realization that this role of the IGF axis is highly conserved or “public” [21,35]. Mutations in the IGF pathway have subsequently been shown to extend lifespan and health span not only in *C. elegans* and *D. melanogaster* but also in rodents and may even contribute to extreme longevity in humans [38,39]. For instance, low levels of free IGF-1 have recently been associated with increased old age survival and better functional status [38,39]. Mutations in the human growth hormone receptor gene, leading to IGF-1 deficiency and dwarfism, may confer exceptional resistance against diabetes and certain cancers [40]. As expected from evolutionary theory, IGF pathway mutations result in animals that are long-lived, have elevated stress resistance to several stressors but are also often associated with fitness tradeoffs such as reduced fecundity or slower development [41,42].

Another pathway that has emerged from model organism research as a major “public” regulator of lifespan is the mechanistic Target of Rapamycin (mTOR) pathway. First characterized in yeast [43], the mTOR pathway is highly conserved, integrating signaling from the IGF pathway with stress response, nutrition and energy sensing [44,45,46]. Decreased mTOR signaling can increase lifespan in *S. cerevisiae*, *C. elegans,* and *D. melanogaster* and in mice [44,45,46,47]. Similar to IGF signaling, mutations in mTOR signaling are often associated with developmental and fitness tradeoffs, such as delayed development, delayed or reduced fecundity or reduced metabolism early in life [48].

The observation that longevity is a highly modifiable trait that appears to be regulated through relatively few evolutionarily conserved (“public”) signaling pathways governing pleiotropic fitness tradeoffs suggests that conserved developmental pathways such as Wnt might be interesting candidates for the identification of novel “virtual” gerontogenes [49]. This is because there are few processes under more stringent evolutionary selection than successful, efficient and rapid completion of developmental programs leading to rapid instantiation of a healthy reproductively competent adult in the minimum time and using the smallest amount of resources possible. Recently, this insight has resulted in a novel class of aging theories and a new approach to identifying pathways involved in longevity assurance and aging [48,50,51].

**Figure 1:**
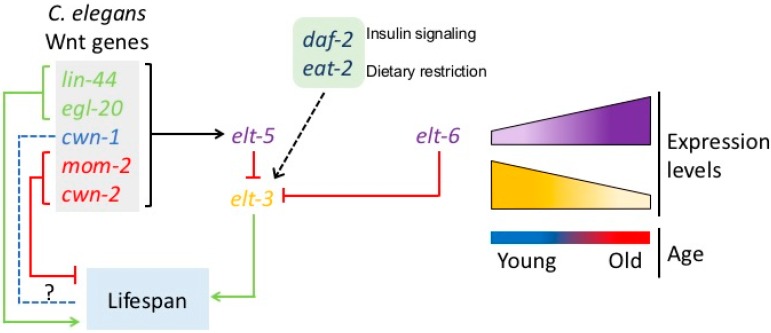
The relationship between *C. elegans* Wnt genes, IGF signaling, dietary restriction and the ELT circuit in aging. Two Wnt genes *lin-44* and *egl-20* extend lifespan while two others *mom-2* and *cwn-2* shorten it. *Cwn-1* did not have a significant effect on lifespan. Wnts increase expression of *elt-5*, which along with *elt-6* has increased expression with age. Both *elt-5* and *elt-6* repress expression of *elt-3* whose expression decreases with age. *Elt-3* is required for lifespan extension through the insulin pathway (*daf-2*) and dietary restriction (*eat-2*).

A key realization was that genes or pathways that are required and tightly regulated during development, may not necessarily have equally tightly regulated functions in adults. Due to the declining force of selection, such pathways may not be so closely controlled (e.g., may not be completely inactivated) during adulthood and may therefore undergo developmental drift, possibly with detrimental consequences for the adult organism. One pathway that shows this type of developmental drift and can be used to modify lifespan was identified in *C. elegans.* The *elt-3/elt-5/elt-6* GATA transcriptional circuit appears to cause aging in *C. elegans*. ELT are a family of GATA transcription factors downstream of Wnt that play an important role during development [50]. Budovskaya et al. were able to show that *elt-5* and *elt-6* expression continues to increase in adults, progressively suppressing expression of *elt-3.* Suppression of *elt-3* is detrimental to lifespan and most interestingly, modification of this circuit can be used to significantly extend lifespan [50]. This circuit therefore represents an example of a pathway that, while required early in life, becomes detrimental late in life (Figure 1). An additional feature of this circuit is that *elt-3* is required for lifespan extension not only through mutation in the IGF axis, but also in a *C. elegans* model of caloric restriction the feeding-deficient *eat-2* worms [50]. Taking these observations together, drift in the *elt-3* circuit links two of the best characterized and conserved lifespan-modifying perturbations [52].

As both *elt-5* and *elt-6* are regulated by Wnt signaling during worm development [7,53,54,55], it is likely that this GATA transcriptional regulation is controlled similarly later in adulthood linking Wnt to this aging related circuit [7,56]. Indeed, all five *C. elegans* Wnts have been reported to be continually expressed in nematodes even after development has been completed [7]. Furthermore, all five Wnt ligands activate expression of *elt-5*, resulting in suppression of *elt-3*, thereby linking drift in the *elt-3/elt-5/elt-6* circuit tightly with the Wnt pathway. However, despite their continued function in adult animals with apparently similar effect on the *elt-3/elt-5* circuit, the effects of the different Wnt ligands on adult lifespan are not identical (Figure 1). While mutations in *mom-2/Wnt* and deletion of *cwn-2/Wnt* resulted in lifespan extension, the opposite was true for mutations in *lin-44/Wnt* and *egl-20/Wnt*. Deletion of *cwn-1/Wnt* did not affect lifespan [7]. As loss of most Wnt genes causes developmental disruptions, inactivation of Wnts in adult wild type N2 animals using RNA interference is used to overcome these developmental phenotypes and explore their pleiotropic longevity effects. Expression of RNAi to *mom-2/Wnt* significantly extended lifespan while RNAi to *lin-44/Wnt* shortened it [7]. It is likely that the complexity in phenotypes is partially due to the fact that, consistent with their function and specificity during development, there is a high degree of tissue specificity in terms of expression amongst the five Wnts during aging. Furthermore, the overall levels and temporal dynamics of expression of each of the Wnt ligands are markedly different. While future work should further explore these complexities and their underlying molecular mechanisms, the authors were able to link Wnt functions back to the regulation of the ELT aging circuit described above [7]. Interestingly, this connection means that both examples for developmental drift that have been reported to date are linked to the Wnt pathway.

Linking Wnt family genes with a transcriptional circuit regulating aging is a major mechanistic advance for the developmental drift theory of aging as this “public” pathway is highly conserved in animals where even sponge genomes contain Wnt genes [5,57]. Wnt expression changes as animals age and aging specific effects have been observed in animals as disparate as flies and mice [2]. Additionally, disruptions in the pathway cause developmental defects and can contribute to human aging related diseases such as osteoporosis and cancer [2]. As one of the few central developmental pathways, Wnts are key candidates for genes with strong early selection, because tight regulation of Wnt activity is required for execution of developmental programs [5]. Due to this well-established early role in development, many Wnt genes have not been extensively studied later in life, and aside from intense study of Wnts in cancer, it is important to note that pleiotropic lifespan and health span effects of post-developmental inactivation of developmental genes are not routinely investigated as part of investigations into developmental signaling pathways.

These findings put the Wnt signaling pathway directly into the developmental drift category of aging pathways, with both a positive and a negative role in aging, which is exactly what would be expected from a pleiotropic aging pathway. As there are several signal transduction pathways downstream of Wnts, and Wnts can often have opposing roles in different tissues [58,59], it is not surprising that different Wnts produce different effects. It can therefore be concluded that Wnts are a key gene family that governs developmental drift and that this may constitute a “public” (applying to disparate species such as *C. elegans*, *Drosophila*, mice and hopefully humans) aging mechanism. Further research into modification of Wnt signaling in adult animals will be an exciting avenue for the identification of novel lifespan and healthspan modifying interventions.

## 4. Connection between Developmental Drift and ROS

Transcriptional targets of *elt-3* include stress response and antioxidant genes, including *sod-3*, the mitochondrial superoxide dismutase involved in detoxification of superoxide in nematode mitochondria. This links the *elt-3/elt-5/elt-6* circuit to free radical detoxification, oxidative stress and redox signaling [50]. Oxidative stress does not seem to be involved in the upstream control of *elt-3*, but *elt-3* mutants are more sensitive to the ROS generating vilogen herbicide paraquat and to heat stress while mutants in *elt-5* are slightly less so [50]. The *elt-3/elt-5/elt-6* circuit may thus be involved in the controlling levels of ROS and oxidative damage. This provides an interesting link between developmental drift and one of the traditional mechanistic damage accumulation theories of aging, the free radical theory of aging (FRTA). According to the FRTA, aging is caused by accumulation of macromolecules that are irreversibly modified by reactions involving reactive oxygen species (ROS) and related chemicals [60,61,62]. Since its inception in 1956, the FRTA has gone through multiple iterations and has given rise to several variants, but the key assumption remains that an important “public” mechanism of aging is age-dependent accumulation of oxidative damage and progressive mitochondrial deterioration, possibly secondary to such oxidative damage [54,55,56]. A detailed discussion is beyond the scope of this review but it is important to note that, there is extensive evidence that oxidative damage is constantly generated *in vivo* and that such damage indeed accumulates with age [63,64,65,66,67]. Furthermore, oxidative damage as well as mitochondrial function or its decline have been implicated in the etiology of many age-dependent diseases and conditions [68]. While there is little doubt that oxidative stress, mitochondrial and metabolic decline contribute to organismal aging, the evidence for a direct, causal role of oxidative damage in the aging process itself is more equivocal with several results in recent years questioning ROS mediated damage as universal cause of aging [5,57,58,59,60].

In parallel with these challenges to core assumptions of the FRTA, there is, however, a growing realization that ROS and ROS products act as signaling molecules, impacting a wide range of processes [26]. ROS, oxidative stress and ROS-mediated damage also play important roles in shaping life-history trajectories by mediating pleiotropic trade-offs between metabolic capacity, reproductive performance, investment into somatic maintenance and potential late-life detriments, like damage accumulation and somatic mutation rate [69]. ROS therefore are not simply the cause of unintended oxidative damage but function also as signaling molecules mediating pleiotropic gene effects.

**Figure 2:**
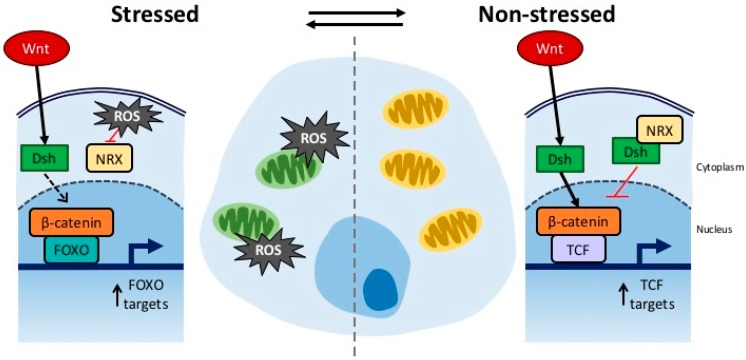
Association of the Wnt pathway with stress responses and aging. Under stress conditions ROS is produced in the mitochondria, blocking the interaction of Nrx with Dsh and releasing Nrx inhibition of Dsh. β-catenin then preferentially interacts with FoxO to activate transcription. When no oxidative stress is present Nrx binds Dsh, and -catenin returns to its normal TCF preference.

A related issue is to what extent ROS are involved or required for successful execution of early developmental programs, in particular those involving Wnt. The Wnt pathway has previously been associated with ROS through a preferential association of β-catenin with the forkhead transcription factor FOXO, rather than TCF, in response to oxidative stress (Figure 2). Moreover, there is extensive evidence that not only β-catenin but several of the major Wnt related signaling pathways are subject to redox regulation (reviewed in [70]). The overall effects of ROS levels on Wnt signaling are complex. For instance, nucleoredoxin (Nrx), a thioredoxin-related protein, can sequester Disheveled under non-stressed conditions, thereby inhibiting Wnt. In response to elevated ROS, nucleoredoxin releases Disheveled, thereby permitting assembly of the activation complex and activating downstream Wnt signaling [71]. Data in both mice and frogs suggest that elevated levels of ROS are, in fact, required for the execution of successful developmental programs. Both early cell fate decisions during development in mice and tail regeneration in tadpoles can be disrupted by antioxidant treatment through mechanisms involving Wnt [72,73]. In adult animals, ROS continues to modulate the activity of several ROS-sensitive factors, including Wnt, in part through partitioning and localization of the limiting β-catenin pool [70,74]. This links Wnt signaling to ROS dependent control of major pro and anti-proliferative as well as stress response pathways, including FOXO and c-Jun. Interestingly, while all five *C. elegans* Wnts intersect with the *elt-3/elt-5* circuit and several Wnt mutations result in either detrimental or beneficial differences in lifespan (see above), there is no evidence that either of these detrimental or beneficial mutations directly impact stress resistance towards acute paraquat challenge or heat shock [7]. Nevertheless, given the central role of β-catenin in coordinating ROS-sensitive transcription factors, including FOXO, an alternative link between ROS and drift in Wnt signaling is through interference with the of the trigger levels at which the effect of elevated ROS switches from promoting proliferation to triggering stress [7]. If Wnt is indeed involved in mediating organismal responses or anti-proliferative/apoptotic pathways [70,74], aberrant Wnt signaling might thus contribute to age-dependent dysregulation of ROS dependent signaling and blunt stress responses, both of which are hallmarks of aging. Detrimental effects of developmental drift in Wnt that are mediated by disruption of redox signaling, resulting in dysregulation of responses to physiological levels of endogenous ROS, might also impact age-dependent disease processes involving ROS and ROS-mediated damage but not show strong effects when tested under acute, non-physiological (lethal) stress challenge. Instead of damage accumulating until something goes wrong, this would be a case of something going wrong, until damage starts to accumulate, leading to new downstream pathology.

Damage accumulation, stress resistance, investment in organismal maintenance and developmental signaling are linked by the recent discovery that stress responses, in particular activation proteostasis due to heat shock, is actively repressed by germ line signaling early during *C. elegans* adulthood [75]. It will be important to investigate links between life history trajectories and damage accumulation dynamics (e.g., oxidative or protein aggregation [76]) under perturbations of the Wnt system. Overall, the intersection of ROS and ROS mediated modifications to macromolecules, early developmental signaling pathways such as Wnt, and of mechanisms controlling life-history trade-offs places Wnt at the intersection between development, developmental drift, lifespan determination, redox signaling and control of life-history trajectory.

## 5. Conclusions: The Potential for Future Translation

Almost a century ago, Osborne glimpsed the plasticity of aging by finding that rats subjected to caloric restriction (CR) experienced delayed and restricted growth, but also lived longer, a finding later confirmed by McCay who more carefully investigated the link between CR, developmental tradeoffs and lifespan [77,78]. The fact that lifespan is a highly modifiable trait has since been confirmed in model organisms using both transgenic approaches and selection experiments [35,79]. Currently, there is interest and progress in developing pharmacological interventions that delay age-dependent disease and decline [80].

Under the developmental drift theory of aging, developmental signaling pathways are the perfect targets in the search for ageing interventions as they are highly conserved and, while advantageous early during development, their effects can be detrimental later in life. As described above, several Wnt ligands have functions adult stem cell homeostasis that are beneficial for the adult organism, but other Wnts can have detrimental effects on longevity. Further, as organisms age there is a decline in homeostatic capacity, a symptom that could be due to loss of Wnts at the stem cell niches leading to fewer stem cells and a loss of organ repair. Overall, inhibition or modulation of Wnt pathways in adult animals is expected to be associated with complex effects on adult humans. As new small molecule cancer treatments are being developed for Wnt-related tumors, it will be interesting to see if these cancer therapies can cause life span extension as well, or if the effects on stem cell homeostasis outweigh the benefits [81].
